# PROTACs for Collateral Degradation: Is It Time to Back Up the Bus?

**DOI:** 10.1021/acsmedchemlett.5c00645

**Published:** 2026-01-12

**Authors:** Zhe Gavin Gao, Kevin Burgess

**Affiliations:** Department of Chemistry, 14736 Texas A & M University, Box 30012, College Station, Texas 77842, United States

**Keywords:** PROTACs, degrader, cyclin, CDK, cell cycle, PRC2, PBAF, HDAC

## Abstract

Off-target effects are usually undesirable in drug development, but for some PROTACs and other degraders, they may be advantageous. Specifically, this can be so when the activities of multiprotein complexes are more important than just the targeted protein of interest.

## Graphical abstract

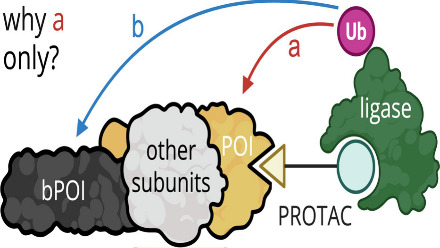

PROTACs comprising linked small molecule fragments for a POI and an E3 ligase, are inherently limited: if there is no known POI ligand, there is no clear-cut design strategy.^1^ Consequently, POI ligands modified to form PROTACs are often enzyme inhibitors. For instance, recent reviews claim 45% of reported PROTACs targets are based solely on kinases.^2^

Protein *complexes* are common in cell biology. *Indirect* degradation of complexed proteins can be envisaged in at least three ways (Figure 1). First, bystander proteins (bPOI) can remain complexed after POI ubiquitinylation and then drawn into the proteosome together with the POI (Figure 1, pathway a). Alternatively, both the POI *and* the bPOI could be ubiquitinated, and both could then be degraded together (Figure 1, path b), or separately if the protein complex dissociates (Figure 1, path c). In some cases, these types of *collateral damage* could be responsible for undesirable off-target effects, but in others, they could enhance impacts of primary degradation events.

**Figure 1:**
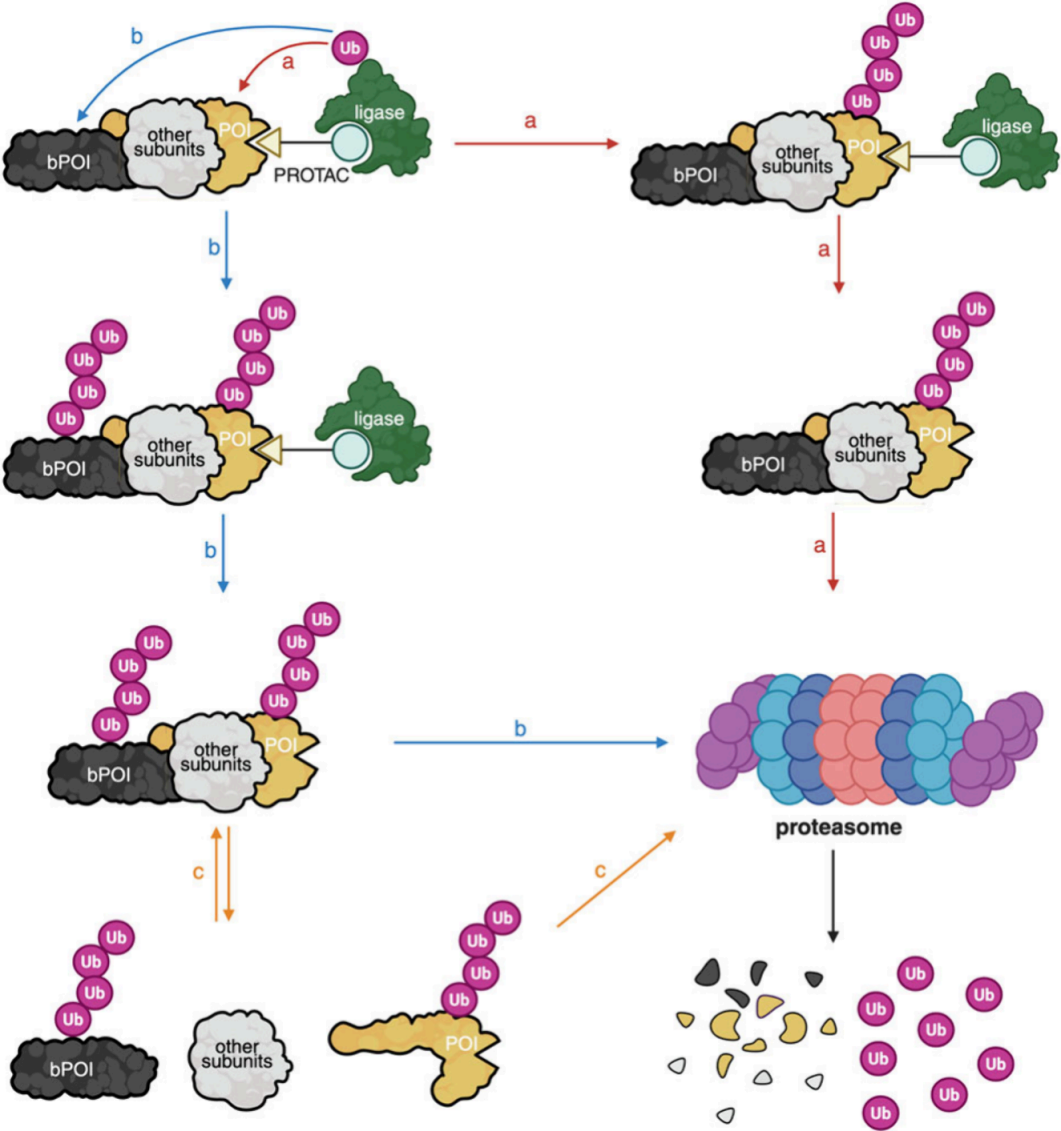
PROTAC could mediate ubiquitinylation of (a) POIs causing degradation of the complex, (b) POIs and bystanders which are then degraded as complexes, and (c) POIs and bystanders which dissociate before degradation.

Complexes of CDKs with cyclins illustrate potential *beneficial collateral damage*. CDKs are critical to cell cycling (Figure 2). CDK inhibitors are prime targets for medicinal chemistry, especially in tumorigenic cells containing enhanced CDK levels, relative to normal cells. Most advanced among these inhibitors are CDK4/6*i*s which have several clinically approved applications.^3,4^ However, tumors tend to acquire immunity to kinase inhibitors^3,5^ limiting their clinical effectiveness to months, then the disease progresses. “Medicinal whack-a-mole” ensues with searches for other CDK targets, beginning with CDK2*i*s, because CDK2 is the next logical target.^6^ CDK2*i*s are likely to be limited by acquired immunity, similarly limiting their clinical efficacy window, so a better strategy is needed.

**Figure 2:**
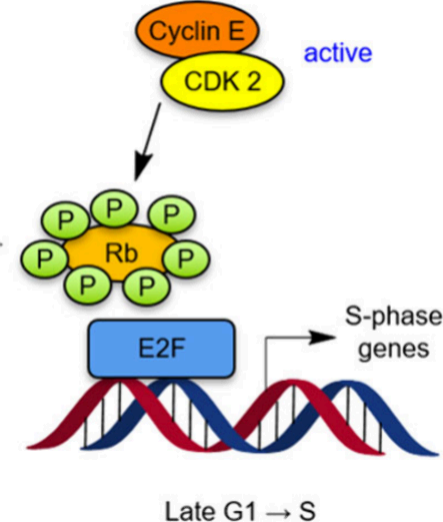
Rb suppresses gene expression by the E2F transcription factor until mitogenic stimuli initiate the production of cyclin D. CDKs 4/6 and accumulation of CDK2•cyclin E lead to pRb and drive the G1/S transition.

CDKs *require* complexation with cyclins for activation in the cell cycle.^7,8^ Cyclins typically have no known small molecule binding sites; therefore, strategies to disable them via inhibition or direct degradation are unknown.

Studies from our laboratories in collaboration^9^ showed CDK2 targeted PROTACs *can also degrade its cyclin E binding partner as collateral damage*. Cancer cells upregulate cyclin E, leading to CDK2-mediated hyperphosphorylation of suppressor retinoblastoma protein (Rb) hence loss of cell-cycle control.^10–12^ Cyclin E is *dispensable* for normal cell replication.^13^ Consequently, simultaneous degradation of CDK2 *and* cyclin E may offer advantages over inhibition of just the kinase and perhaps postpone acquired immunity. Figure 3 shows a crystal structure of CDK2•cyclin E^14^ highlighting surface exposed Lys residues on the cyclin, some of which may be available for ubiquitylation via Figure 1b or c, but it is unclear which mechanisms are applicable at this stage.

**Figure 3:**
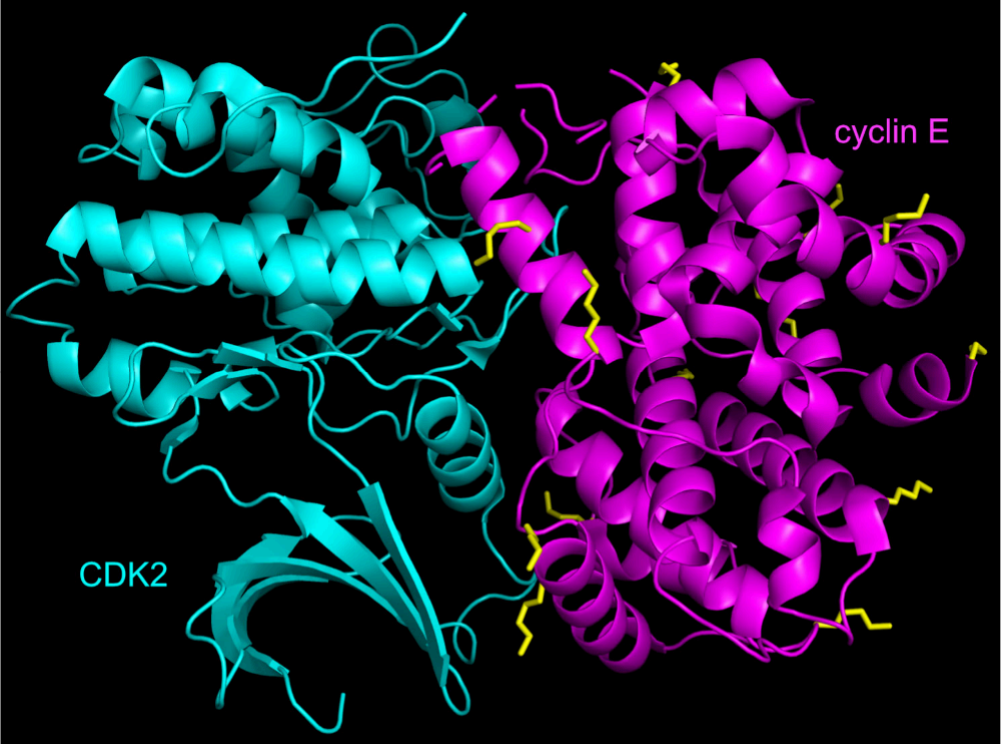
Crystal structure of CDK2/cyclin E complex (PDB 7KJS).^14^ Surface exposed lysine side chains of cyclin E are shown to illustrate that there are many of these to which ubiquitin could be transferred via paths b and c in Figure 1.

PROTACs developed for other CDKs also can degrade their cyclin partners.^15^ This has not been explored for all CDK PROTACs reported so far but was demonstrated in several cases. Thus, PROTACs of CDKs 4/6, 8, and 9 have been shown to degrade their corresponding cyclin partners D,^16^ C,^17^ and T,^18,19^ respectively. These are all oncology targets, and some also have other potential applications, *eg* CDK9•cyclin T is relevant to treatment of patients with HIV.^20^

Exploration of collateral damage reports is exceptional for CDK degraders, probably because localized off-target effects are not routinely assayed. For important cases, particularly for advanced clinical candidates, if this possibility has not been checked, then it may be worthwhile to do so. Currently it is hard to be sure if localized off-target effects have been evaluated it is difficult to discern *not checked* from *checked but not reported*, and raw data from pertinent clinical trials is often unavailable. Specifically, it would be interesting to know whether collateral damage occurs for the PROTACs: BTX-9341 (Biotheryx, NCT06515470), targeting CDK4/6, in phase I for advanced or metastatic breast cancer;^21^ and NKT-3964 (Nikang Therapeutics, NCT06586957), targeting CDK2 in phase I for advanced and metastatic solid tumors.

It is difficult to search the literature for codegradation exclusively within complexes, but the examples described here seem to be prevalent. We are struck that potentially beneficial cases tend to feature the cell cycle, including PRC complexes, described below, since these directly impact cyclin D and E.^22^ Prevalence of the cell cycle could be a coincidence or might indicate cyclins are potentially valuable *silent targets* for degraders because there are no PROTACs targeting cyclins. This may not be so in other cases.

PRC2 (Figure 4) is an epigenetic transcription modulator. It regulates gene expression via histone modification.^23^ Biological outcomes of these molecular events are stem cell maintenance, epithelial to mesenchymal transitions, and DNA repair.^24^ Thus, there are opportunities to modulate activities of PRC complexes using PROTACs, and some of these are being exploited.

**Figure 4:**
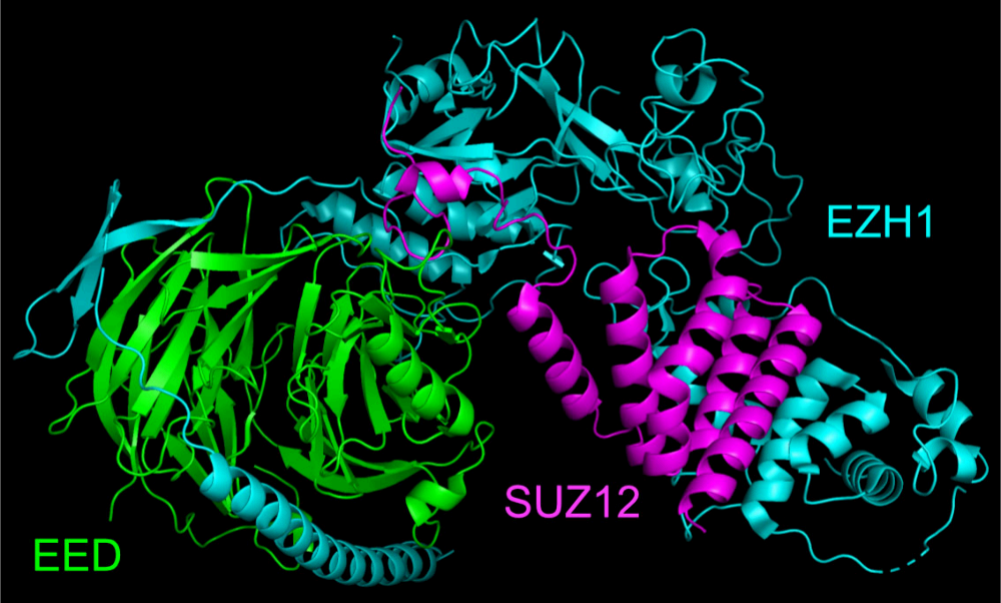
Crystal structure of PRC2 complex (7TD5).^23^

PROTACs have been developed to target EZH2 ^25–28^ (a histone methyl transferase), and EED^29–31^ (a transcription factor inhibitor). For both, collateral damage of SUZ12 and off-target EED or off-target EZH2 has been observed. The PROTAC for EZH2, AXT-1003 (Axter Therapeutics, structure undisclosed, NCT06484985), is most advanced, being in Phase I trials for relapsed Non-Hodgkin Lymphomas.

There are three major types of PRC complexes 1, 2, and -DUB.^32^ PRC1 is phosphorylated by CDK cyclin complexes during mitosis, hence is essential for proper cell division during cytokinesis, the final step of the cell cycle.^33^ PROTACs targeted to EED in PRC1 degrade *two* components: BMI1 and RING1B^34,35^ (these are not found in PRC2; there is no published PRC1 structure containing EED; hence it cannot be shown here).

NCoR, CoREST, Sin3, NuRD, MiDAC and ELM-SANT are HDAC-containing repressive complexes.^36^ HDAC degradation can inflict collateral damage on *many* components of each of those complexes.^36^ Degradation of all of them is unlikely to be beneficial for the treatment of any particular disease state. Consequently, searches for totally selective HDAC inhibitors are of current interest, particularly for specific isoforms within the same class.^37^ Effects of precisely targeted HDACs in the future could be enhanced by off-target collateral damage.

PBAF is a complex that dictates gene expression by remodeling chromatin structures but does not contain an HDAC component. It consists of a motor subunit SMARCA4 and 11 auxiliary subunits. A PROTAC (based on a SMARCA ligand found by HTS) also degraded at least one of these subunits, PBRM1, though it is unclear what others were tested for codegradation.^38^

A potentially general approach to specific complexes for gene expression is to use target DNA sequences as degrader warheads. There is at least one example of this involving collateral damage: targeting NRF2•MafG heterodimer with a PROTACs based on a nucleotide 21-mer, was used to degrade the whole complex simultaneously.^39^ A limitation is that a transfection strategy is required, which is not ideal for *in vivo* applications; that may mean such degraders are only useful as elaborate cellular probes.

Collateral damage within a complex might *not* be desirable for other aspects of cell biology. Even for PRC2 it is not totally clear it is since modifications of EZH2,^25,26^ SUZ12, or EED^30,31^ are known to drive aberrant *hypermethylation* of H3K27 in several cancers. In other cases, the onus is on proving collateral damage is beneficial or at least benign. Overlaid on this, testing for degradation of all components of multicomponent complexes (*cf*. for PBAF there are 11; see above) is arduous, and the biomedicinal consequences are even harder to determine. Nevertheless, beneficial effects of collateral damage are expected to continually emerge.

## Abbreviations

PROTAC: Proteolysis-targeting chimera
Ub: Ubiquitin
POI: Protein of interest
CDK: Cyclin-dependent kinase
Rb: Retinoblastoma protein
PRC: Polycomb Repressive Complex
EED: Embryonic Ectoderm Development
EZH1/2: Enhancer of zeste homologue 1/2
SUZ12: Suppressor of zeste 12 protein
PR-DUB: Polycomb repressive deubiquitinase
HIV: Human immunodeficiency virus
PBAF: Polybromo associated BRG1 associated Factor
NRF2: Nuclear factor erythro 2
MafG: MAF bZIP transcription factor G
HTS: High throughput screening
